# Nef Obtained from Individuals with HIV-1 Vary in Their Ability to Antagonize SERINC3- and SERINC5-Mediated HIV-1 Restriction

**DOI:** 10.3390/v13030423

**Published:** 2021-03-06

**Authors:** Zita Kruize, Ad C. van Nuenen, Stan W. van Wijk, Arginell F. Girigorie, Karel A. van Dort, Thijs Booiman, Neeltje A. Kootstra

**Affiliations:** Department of Experimental Immunology, Amsterdam UMC, Amsterdam Infection & Immunity Institute, University of Amsterdam, 1105 AZ Amsterdam, The Netherlands; z.kruize@amsterdamumc.nl (Z.K.); a.c.vannuenen@amsterdamumc.nl (A.C.v.N.); s.w.vanwijk@amsterdamumc.nl (S.W.v.W.); a.f.girigorie@amsterdamumc.nl (A.F.G.); k.a.vandort@amsterdamumc.nl (K.A.v.D.); tbooiman@halix.nl (T.B.)

**Keywords:** HIV-1, Nef, SERINC3, SERINC5, amino acid variations, naturally occurring mutations, disease progression

## Abstract

Nef is a multifunctional viral protein that has the ability to downregulate cell surface molecules, including CD4 and major histocompatibility complex class I (MHC-I) and, as recently shown, also members of the serine incorporator family (SERINC). Here, we analyzed the impact of naturally occurring mutations in HIV-1 Nef on its ability to counteract SERINC restriction and the clinical course of infection. HIV-1 Nef sequences were obtained from 123 participants of the Amsterdam Cohort Studies and showed multiple amino acid variations and mutations. Most of the primary Nef proteins showed increased activity to counteract SERINC3 and SERINC5 as compared to NL4-3 Nef. Several mutations in Nef were associated with either an increased or decreased infectivity of Bal26-pseudotyped HIV-1 produced in the presence of SERINC3 or SERINC5. The 8R, 157N and R178G Nef mutations were shown to have an effect on disease progression. Survival analysis showed an accelerated disease progression of individuals infected with HIV-1 carrying arginine or asparagine at position 8 or 157 in Nef, respectively, or the R178G Nef mutation. Here, we observed that naturally occurring mutations in Nef affect the ability of Nef to counteract SERINC3- and SERINC5-mediated inhibition of viral infectivity. The majority of these Nef mutations had no significant effect on HIV-1 pathogenesis and only the 8R, 157N and R178G mutations were associated with disease course.

## 1. Introduction

Nef is a 27–32-kilodalton (kDa) accessory protein encoded by HIV-1 and other primate lentiviruses. Nef is a multifunctional viral protein that has the ability to downregulate cell surface molecules, including CD4, major histocompatibility complex class I (MHC-I) and T-cell receptor, via clathrin-dependent endocytosis and subsequent lysosomal degradation [1]. Nef’s ability to internalize CD4 and MHC-I from an infected cell surface allows HIV-1 to evade antibody-dependent cellular cytotoxicity (ADCC) and cytotoxic T lymphocytes (CTLs) [2,3,4,5]. Nef can also alter the activation state of T cells and macrophages and perturbs the actin cytoskeleton by engaging with cellular kinases [6,7,8].

One of the most conserved but poorly understood functions of Nef is the enhancement of progeny virion infectivity. Nef-mediated enhancement of viral infectivity was recently shown to be due, in part, to its ability to counteract members of the serine incorporator family (SERINC). SERINC proteins are host restriction factors, of which SERINC5 was shown to be the most potent one [9,10]. SERINC5 can be incorporated in the membrane of progeny virions, which subsequently inhibits infection of new target cells at the level of membrane fusion [11]. HIV-1 Nef has been shown to prevent SERINC5 incorporation into the virion and thus counteracts the SERINC5-mediated restriction. Nef interacts with SERINC5 on the cell surface, and upon internalization, SERINC5 is targeted for lysosomal degradation [12]. Several Nef mutations involved, among others, in myristoylation, dimerization and interaction with the clathrin- and adaptor protein complex 2 (AP-2) are reported to impair the Nef function [13,14,15,16]. Although a role of SERINC in HIV-1 replication has clearly been demonstrated, the impact of naturally occurring mutations in HIV-1 Nef on its ability to counteract SERINC restriction and the clinical course of infection is not well known. In this study, we assessed if naturally occurring, previously described genetic variation and mutations in the identified binding sites and mutations of HIV-1 Nef have an effect on the ability to counteract SERINC3 and SERINC5. Moreover, we determined the effect of these mutations on disease progression during the natural course of infection in the Amsterdam Cohort Studies.

## 2. Materials and Methods

### 2.1. Study Population

The study population consisted of men having sex with men (MSM) enrolled in the Amsterdam Cohort Studies (ACS) on the natural history of HIV-1 infection between October 1984 and February 1988. HIV-1 Nef sequences were obtained from 123 participants with known date of seroconversion and documented AIDS diagnosis or follow-up of at least 7 years without an AIDS diagnosis [17]. All participants in this study were infected with subtype B HIV-1.

### 2.2. Ethics Statement

The ACS has been conducted in accordance with the ethical principles set out in the Declaration of Helsinki, and written informed consent was obtained prior to data collection. The study was approved by the Amsterdam Medical Center institutional medical ethics committee (MEC 07/182, 20 August 2007).

### 2.3. HIV-1 Nef Sequencing

Clonal HIV-1 variants or bulk isolates were obtained as described previously [18] and total DNA was isolated using the L6 isolation method. Nef was amplified using a nested PCR with the outer primers Nef-1-fw (5′-AGCCATAGCAGTAGCTGAGG-3′) and Nef-1-rev (5′-GCTTATATGCAGGATCTGAGG-3′) and the inner primers Nef-2-fw (5′-AGCTTGTAGAGCTATTCGCCACA-3′) and Nef-2-rev (5′-AGCAAGCTCGATGTCAGCAG-3′). Nef PCRs were performed using Taq polymerase (Promega, Madison, WI, USA) in the presence of 2 mM MgCl2 using the following amplification cycles: 2 min at 95 °C, 35 cycles of 30 s at 95 °C, 30 s at 55 °C, 2 min at 72 °C, followed by a 10-min extension at 72 °C and subsequent cooling to 4 °C. PCR products were purified using EXOSAP-IT reagent (USB, Cleveland, OH, USA) and sequenced using an ABI prism Big Dye Terminator v1.1 Cycle Sequencing Kit (Applied Biosystems, Foster City, CA, USA) using the nested PCR primers. Sequences were analyzed on the Applied Biosystems/Hitachi 3130 xl Genetic Analyzer (Foster City, CA, USA). DNA sequences were translated and analyzed with the BioEdit program (BioEdit v 7.0.5, Tom Hall, Ibis Therapeutics, Carlsbad, CA, USA).

### 2.4. Plasmids

SERINC3- and SERINC5-expressing constructs were constructed by cloning complementary DNA (cDNA) of SERINC3 and SERINC5 into a HIV-1-based lentiviral expression vector in which expression was driven by the human Elongation Factor 1 alpha promotor, using the In-Fusion^®^ HD Cloning kit as described by the manufacturer (Clontech^®^ Laboratories, Inc., Mountain View, CA, USA). Sanger sequencing demonstrated that the cloned cDNA matched the reference sequence (accession number NM_001174072.2 for SERINC5 and NM_006811 for SERINC3). Constructs expressing the HIV-1 Nef protein were constructed as follows: Nef from primary HIV-1 isolates from individuals participating in the Amsterdam Cohort Studies and NL4-3 were amplified by PCR and cloned in pcDNA3.1 (Invitrogen, Carlsbad, CA, USA) using the In-Fusion^®^ HD Cloning kit as described by the manufacturer (Clontech^®^ Laboratories, Inc., Mountain View, CA, USA). The S163C, R178G and double mutants were created from NL4-3 Nef with site-directed mutagenesis.

### 2.5. Cell Lines

HEK293T cells (ATCC® CRL-3216™)were cultured in Dulbecco’s Modified Eagle Medium without HEPES (DMEM) (Lonza, Basel, Switzerland) supplemented with 10% (*v/v*) inactivated fetal calf serum (FCS; HyClone, Cytiva, Marlborough, MA, USA), penicillin (100 U/mL) and streptomycin (100 µg/mL) (Invitrogen, Carlsbad, CA, USA) and maintained in a humidified 10% CO_2_ incubator at 37 °C. U87/CD4^+^-CCR5^+^ cells (NIH AIDS reagent program) [19] were cultured in Iscove’s modified Dulbecco medium (Lonza, Basel, Switzerland) supplemented with 10% (*v/v*) inactivated FCS, penicillin (100 U/mL) and streptomycin (100 µg/mL), treated once a month with G418 (Sigma-Aldrich St. Louis, MO, USA) and maintained in a humidified 5% CO_2_ incubator at 37 °C.

### 2.6. Virus Production

Bal26-pseudotyped NL4-3.Luc.R-E- luciferase reporter virus, for single-cycle replication assays, was produced by transfection of pNL4-3.Luc.R-E- [20,21,22] with pEnv Bal26 in HEK293T cells. The luciferase reporter virus was produced in HEK293T cells co-transfected in a 6-well plate with plasmids expressing Nef and SERINC3 or SERINC5 alone or in combination. In a 6-well plate, 7.18 µg of pNL4-3.Luc.R-E-, 2.51 µg of pEnv Bal26, 2.51 µg of plasmid expressing Nef or empty pcDNA3.1 vector (Invitrogen, Carlsbad, CA, USA) and 2.51 µg of plasmid expressing SERINC3, SERINC5 or empty lentiviral vector were co-transfected. Transfections were performed using the calcium phosphate method. In short, plasmid DNA was diluted in 0.042 M HEPES containing 0.2 M CaCl_2_ and subsequently mixed with an equal volume of 2× HEPES-buffered saline pH 7.2, incubated at room temperature for 15 min and added to the culture medium. After 24 h incubation in a humidified 3% CO_2_ incubator at 37 °C, the culture medium was replaced and cultures were continued at 10% CO_2_ at 37 °C. Virus was harvested at 72 and 96 h after transfection and passed through a 0.22-µm filter.

### 2.7. Flow Cytometry

HEK293T cells were cultured in 6-well plates and co-transfected with plasmids expressing Nef and SERINC3 or SERINC5 as described above. Cells were harvested with phosphate buffered saline (PBS; Lonza, Basel, Switzerland) supplemented with 10mM EDTA (Sigma-Aldrich St. Louis, MO, USA). HEK293T cells were stained with anti-SERINC3 (1:500, Abcam, Cambridge, UK) and anti-SERINC5 (4 µg/mL, Abcam, Cambridge, UK) for 30 min at 4 °C in the dark. Subsequently, cells were washed twice with PBS supplemented with 0.5% bovine serum albumin (BSA; Sigma-Aldrich St. Louis, MO, USA) followed by staining with an allophycocyanin (APC)-conjugated goat anti-rabbit (1:400; Invitrogen, Carlsbad, CA, USA) for 30 min at 4 °C in the dark. Subsequently, cells were washed again with PBS supplemented with 0.5% BSA and fixed using 250 mL of BD CellFIX (BD Biosciences, Franklin Lakes, NJ, USA). Fluorescence was measured with the FACSCanto II analyzer (BD Biosciences, Franklin Lakes, NJ, USA). The geometric mean of the fluorescence intensity of APC was determined using FlowJo 10 (TreeStar, Ashland, OR, USA).

### 2.8. Western Blot Analysis

HEK293T cells were cultured in 6-well plates and transfected with plasmids expressing Nef as described above. Two days post-transfection, HEK293T cells were lysed in RIPA-buffer (150 mM NaCl, 1% Triton X-100, 0.5% sodium deoxycholate, 0.1% SDS, 50 mM Tris, pH 8.0) containing Complete EDTA-free protease inhibitor (Roche, Basel, Switserland). After adding NuPAGE LDS 4× sample buffer (Invitrogen, Carlsbad, CA, USA) and 0.1 M dithiothreitol (DTT; Invitrogen, Carlsbad, CA, USA), samples were heated at 95 °C for 10 min. Proteins were separated by SDS-PAGE (NuPAGE 4–12% Bis-Tris precast gel and MES SDS running buffer (Invitrogen, Carlsbad, CA, USA) and transferred to a nitrocellulose membrane (Protran, Schleicher and Schuell, Dassel, Germany) using NuPAGE transfer buffer (Invitrogen, Carlsbad, CA, USA). After blocking for 2 h with PBS containing 5% Protifar (Nutricia, Schiphol, The Netherlands) and 0.5% BSA, the blot was incubated with anti-c-Myc antibody (1∶5000; Calbiochem, San Diego, CA, USA) and anti-β-actin antibody (1∶200; Santa Cruz Biotechnology, Santa Cruz, CA, USA). IRDye 800CW conjugated goat anti-mouse IgG (1∶15000, LI-COR, Lincoln, NE, USA) and IRDye 680LT conjugated donkey anti-goat IgG (1∶15000, LI-COR) were used as secondary antibodies to visualize expression using the proteins.

### 2.9. Viral Infection

To analyze the effect of SERINC3, SERINC5 and the primary Nef proteins on HIV-1 replication, U87 CD4+CCR5+ cells were inoculated with NL4-3 luciferase Bal26-pseudotyped single-round reporter viruses produced in the presence or absence of Nef alone or in combination with SERINC3 or SERINC5. Equal amounts of reporter viruses, as determined by an in-house p24 ELISA, were used to inoculate the target cells [23]. Luciferase activity was determined, as a measure of viral replication, 48 h post-infection by adding 25 µL substrate (0.83 mM ATP, 0.83 mM d-luciferin (Duchefa, Haarlem, The Netherlands), 18.7 mM MgCl_2_, 0.78 µM Na_2_H_2_P_2_O_7_, 38.9 mM Tris pH 7.8, 0.39% (*v/v*) glycerol, 0.03% (*v/v*) Triton X-100 and 2.6 µM dithiothreitol) directly to the culture medium. Luminescence was measured for 1 s per well using a luminometer (Berthold, Bad Wildbad, Germany). The infectivity of the virus produced in the presence of the primary Nef and SERINC3/5 was corrected for the infectivity of the virus produced in the presence of the same primary Nef and the relevant empty vector. The ability of the primary Nef proteins to counteract SERINC3/5 was expressed relative to NL4-3 Nef (fold change).

### 2.10. Statistical Analysis

Kaplan–Meier and Cox proportional hazard analyses were performed to study the relation between naturally occurring mutations in Nef and disease progression. The following endpoints were considered for analysis: AIDS-defining events including CD4+ T cell counts below 200 cells/µL according to the 1993 Center for Disease Control (CDC) definition and AIDS-related death. Individuals who started effective combination antiretroviral therapy (cART) or who were lost to follow-up were censored. Student’s *t*-test or the Mann–Whitney U test was used to compare Nef activity of primary Nef from individuals with HIV-1 and wild-type NL4-3 Nef and Nef with and without naturally occurring mutations. Spearman’s correlation was used to calculate the correlation between SERINC3 and SERINC5. Statistical analyses were performed using IBM SPSS Statistics for Windows v.25 (IBM, Armonk, NY, USA).

## 3. Results

### 3.1. Naturally Occurring Variation in Nef

**Figure 1:**
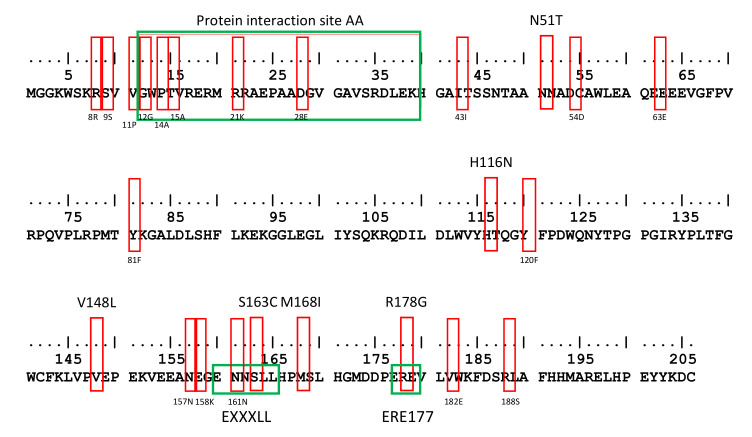
Amino acid variation in Nef. HIV-1 consensus B Nef. The red boxes indicate previously identified amino acid polymorphisms [14,25,26]. The green boxes highlight known binding sites of Nef in which naturally occurring variations and amino acid polymorphisms occur.

Recent reports demonstrated that three specific sites in Nef, the amino acids 12 to 39, the ExxxLL motif (S163C) and the AP-2 binding site (R178G), were identified to be involved in Nef function [14,15,16,24] (Figure 1). In addition, multiple amino acid variations in Nef (subtype B HIV-1) were identified to play a role in SERINC3 and SERINC5 antagonism [14,25,26] (Figure 1) including 8R, 9S, 11P, 12G, 14A/P/S, 15A, 21K/R, 28E, 43I, N51T, 54D, 63E, 81F, H116N, 120F, V148L/X, 157N, 158K, 161N, M168I/X, 182E and 188S, of which some specifically affected SERINC3 (8R, 9S, 11P, 12G, 14A, 15A, 21K, 28E, 43I, 182E and 188S) or SERINC5 (14S, 21R, 54D, 63E, 81F, 120F, 157N and 158K) [26]. We analyzed primary Nef proteins obtained from 123 individuals with HIV-1 participating in the Amsterdam Cohort Studies for amino acid changes at the previously described positions and regions as compared to consensus B (Supplementary Table S1). In 46.3% (57/123) of the patients, insertions of amino acids in the AA 12–39 region were observed, of which 63.2% had insertions of 1–4 amino acids and 36.8% had insertions of 5–16 amino acids (Table 1). In addition, changes in the number of positively charged amino acids were observed in 55.3% (68/123) of the patients, of whom 42.6% showed an increase and 57.4% showed a decrease in positively charged amino acids (Table 1). The S163C mutation in the ExxxLL motif was observed in 39.8% (49/123) of the patients, while the R178G mutation, located in the AP-2 binding site, was observed in 17.9% (22/123) of the patients. The previously described polymorphisms (8R, 9S, 11P, 12G, 14A/P, 15A, 21K/R, 28E, 43I, N51T, 54D, 63E, 81F, H116N, 120F, V148L/X, 157N, 158K, 161N, M168I/X, 182E and 188S) were observed at different frequencies and are shown in Table 1. In the majority of the Nef sequences, a combination of these mutations was observed (Supplementary Table S1). For instance, a change in the number of positively charged amino acids was often observed in combination with an increased length of the AA 12–39 region (30.1%), in combination with the S163C mutation in ExxxLL (22.8%) or combined with both an increased length and the S163C mutation (9.8%).

### 3.2. Effect of Naturally Occurring Amino Acid Variations on Nef Activity to Counteract SERINC3 and SERINC5

**Figure 2:**
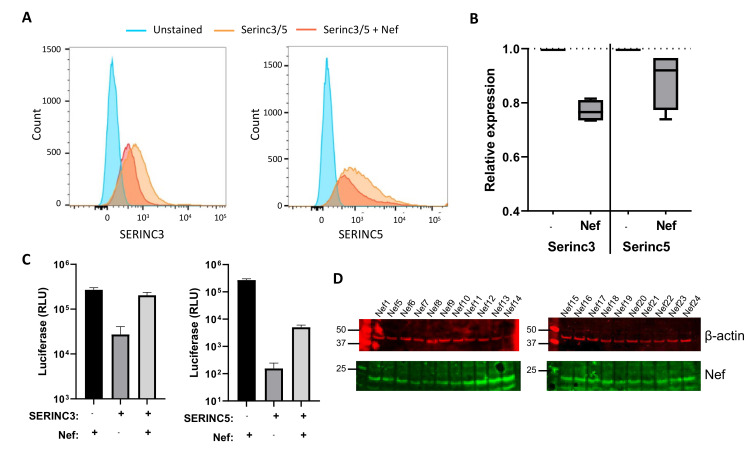
(**A**) Downregulation of serine incorporator 3 (SERINC3) and SERINC5 membrane expression by HIV-1 Nef. HEK293T cells were co-transfected with plasmids expressing Nef and SERINC3 or SERINC5 or in combination with a relevant empty vector. The surface expression of SERINC3 and SERINC5 (orange) or in combination with HIV-1 Nef (red) was determined by flow cytometry as the geometric mean of fluorescent intensity. Background fluorescent (blue) control was included. (**B**) Downregulation of SERINC3 and SERINC5 by Nef as shown as relative expression as compared to the control of four independent experiments (*p* < 0.05). (**C**) The effect of SERINC3 (left panel) and SERINC5 (right panel) on HIV-1 infectivity: Infectivity of Bal26-pseudotyped HIV-1 produced in 293T cells overexpressing Nef (black bar), SERINC3 or SERINC5 (dark gray bar) and SERINC3 or SERINC5 in combination with Nef (light gray bar) in U87 CD4+CCR5+. Infectivity was determined by luciferase activity in relative light units (RLU). Mean ± SD are shown. (**D**) Expression of Nef from pcDNA3.1 encoding primary Nef proteins and β-actin levels were determined by Western blotting.

To assess the ability of HIV-1 Nef to counteract the SERINC3- and SERINC5-mediated inhibition of viral infectivity, SERINC3 and SERINC5 were overexpressed in HEK293T cells, either alone or combined with HIV-1 Nef. Subsequently, the surface expression of SERINC3 and SERINC5 was determined. We observed that the geometric mean of fluorescence intensity for SERINC3 and SERINC5 decreased significantly (*p* < 0.05) in the presence of HIV-1 Nef, indicating a decrease in the surface expression of SERINC3 and SERINC5 by HIV-1 Nef (Figure 2A,B). The infectivity of Nef-deficient HIV-1 produced in the presence of SERINC3 or SERINC5 was reduced (Figure 2C). When a Nef protein was co-expressed during virus production, the infectivity was recovered (Figure 2C).

**Figure 3:**
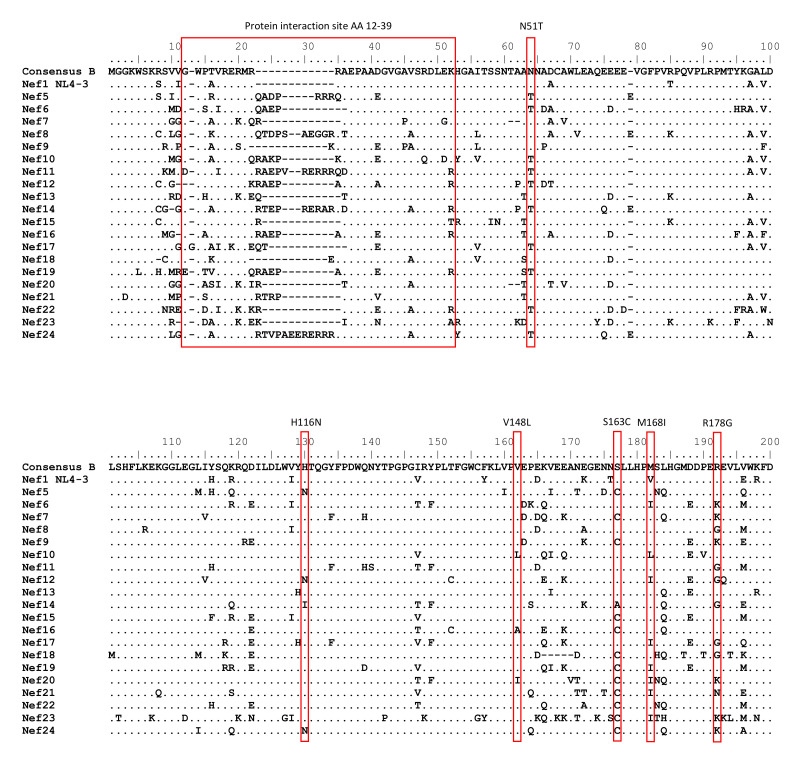

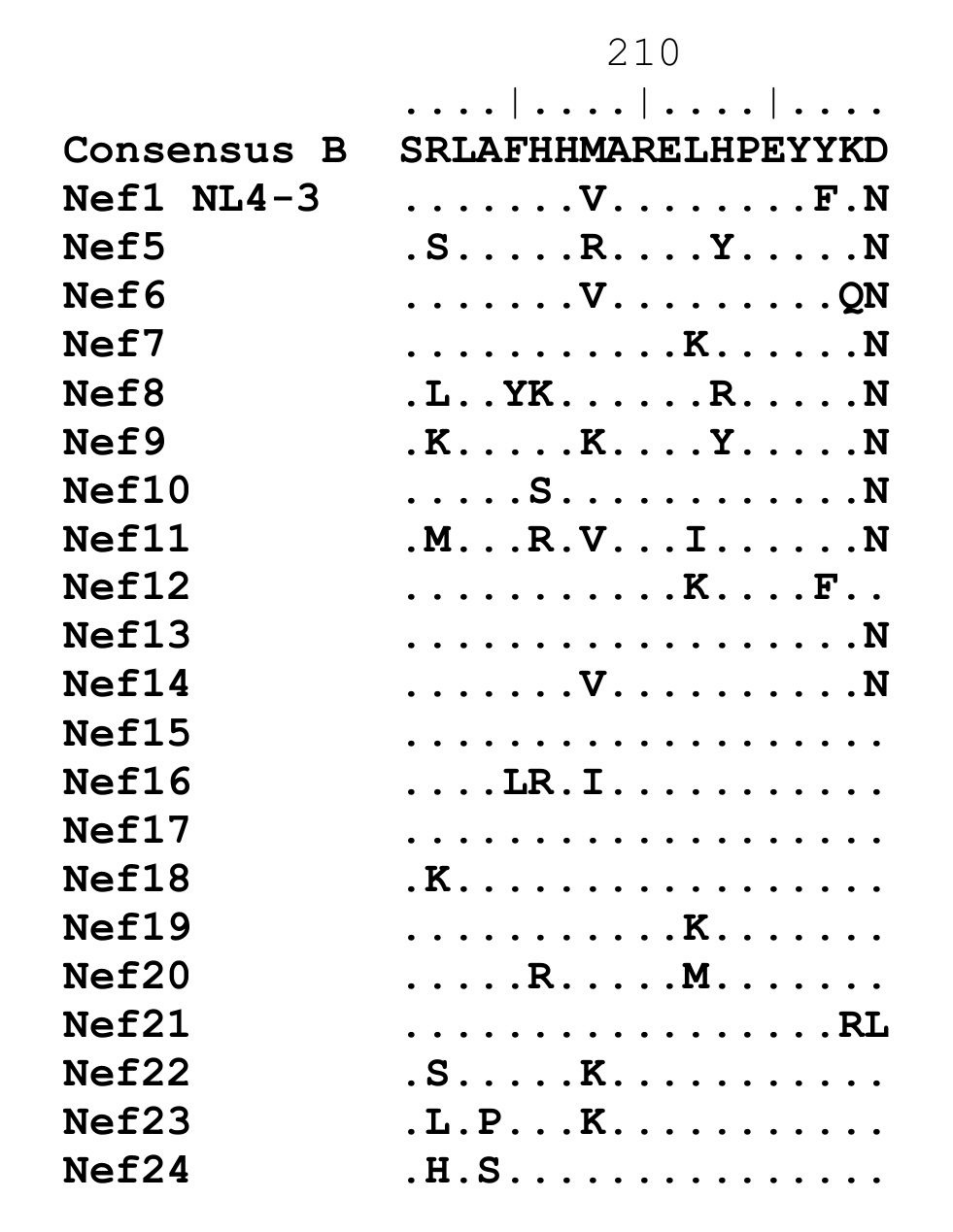
Amino acid variation in Nef proteins obtained from individuals with HIV-1. HIV-1 consensus B Nef, NL4-3 Nef (Nef1) and Nef proteins from patients with HIV-1 (Nef5-24). Red boxes indicate some of the previously described amino acid polymorphisms [14,25,26] and binding sites for reference.

We selected 20 primary Nef proteins from individuals with HIV-1 participating in the Amsterdam Cohort Studies that show amino acid variation in the AP-2 binding site, the ExxxLL motif, changes in the number of positively charged amino acids and length of the protein interaction site consisting of the amino acid residues 12–39 (AA 12–39) and contain the previously identified amino acid polymorphisms [14,25,26] (Figure 3). The primary Nef proteins were cloned into expression plasmids and Nef expression by these constructs was demonstrated by Western blotting (Figure 2D). The effect of naturally occurring mutations in patient Nef proteins on SERINC3- and SERINC5-restricted HIV-1 infectivity (Bal26-pseudotyped single-round luciferase reporter virus) was determined in U87 CD4+CCR5+ cells.

**Figure 4:**
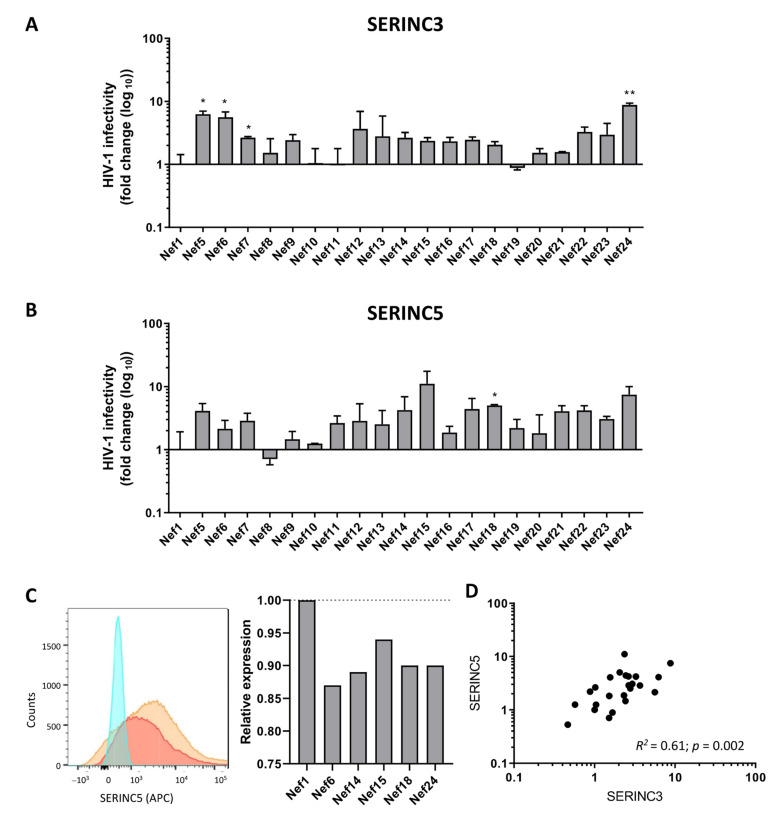
Effect of naturally occurring amino acid variations on infectivity of Bal26-pseudotyped HIV-1. Infectivity of Bal26 Nef-deficient single-round luciferase reporter virus produced in 293T cells overexpressing SERINC3 (**A**) or SERINC5 (**B**) in the presence of wild-type NL4-3 Nef (Nef1) or Nef proteins obtained from individuals with HIV-1 (Nef5-Nef24). The infectivity of the virus produced in the presence of the Nef and SERINC3/5 was corrected for the infectivity of the virus produced in the presence of the same primary Nef and an empty vector. Virus infectivity is expressed relative to the virus produced in the presence of NL4-3 Nef (fold change). Means ± SD of three independent experiments are shown. Unpaired two-tailed *t*-test (* *p* < 0.05; ** *p* < 0.01). (**C**) Downregulation of SERINC5 membrane expression by primary Nef proteins was determined by flow cytometry (Left panel—orange: Nef1; red: Nef24; blue: background fluorescent control). Downregulation of SERINC5 membrane expression by primary Nef (Nef6, 14, 15, 18, and 24) relative to NL4-3 Nef (Nef1) (right panel). (**D**) Correlation between the ability of the primary Nef proteins to counteract SERINC3 and SERINC5.

We showed that the infectivity of Bal26-pseudotyped HIV-1 produced in the presence of primary Nef proteins in combination with SERINC3 or SERINC5 displayed considerable variation in infectivity (Figure 4), but the majority of the primary Nef proteins showed increased Nef activity as compared to wild-type HIV-1 NL4-3 Nef (Nef1). A significant increase in infectivity of HIV-1 produced in the presence of SERINC3 was observed for Nef5, Nef6, Nef7 and Nef24 (Figure 4A), and a trend was observed for Nef9, Nef14, Nef15, Nef16, Nef17, Nef18 and Nef22 (Figure 4B). Nef18 was able to significantly increase the infectivity of HIV-1 in the presence of SERINC5 (Figure 4B), and a trend was observed for Nef5, Nef7, Nef15, Nef17, Nef21, Nef22, Nef23 and Nef24 (Figure 4B). For a selection of primary Nef proteins, an additional flowcytometry analysis was performed to demonstrate downregulation of SERINC5 membrane expression. An increased ability of SERINC5 downregulation was observed for the primary Nef proteins in comparison to Nef1 (Figure 4C), which is in agreement with the increased infectivity of HIV-1 produced in the presence of SERINC5 and primary Nef. Moreover, a significant correlation of the activity of primary Nef proteins to counteract SERINC3 and SERINC5 was observed (*R*^2^ = 0.61; *p* = 0.002) (Figure 4D).

### 3.3. Association between Naturally Occurring Mutations in Nef and the Ability to Counteract SERINC3 and SERINC5

**Figure 5:**
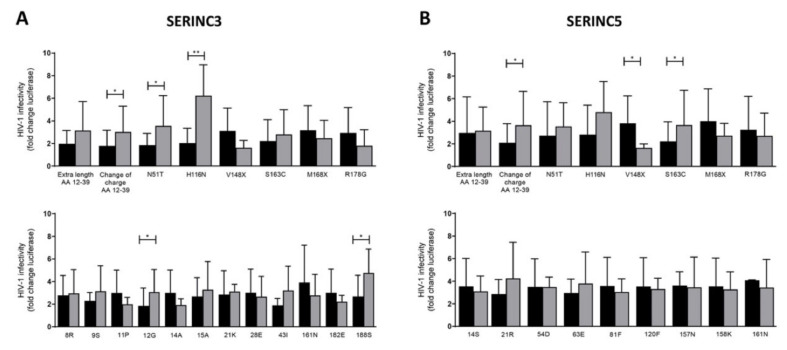
Effect of naturally occurring mutations in Nef on HIV-1 infectivity. The ability of primary Nef proteins that do or do not contain the indicated amino acids or mutations to counteract (**A**) SERINC3- or (**B**) SERINC5-mediated HIV-1 restriction was compared. Virus infectivity is expressed relative to the virus produced in the presence of NL4-3 Nef (fold change of three independent experiments). Means ± SD of Nef proteins containing the indicated amino acid or mutation are given. Mann–Whitney U test (* *p* < 0.05; ** *p* < 0.01). Black bar: indicated amino acid or mutation is absent; grey bar: indicated amino acid or mutation is present.

Next, we determined whether changes in the length and charge of the protein interaction site and naturally occurring Nef mutations were associated with changes in Nef activity to counteract SERINC3 and SERINC5 (Figure 5; all data are shown in Supplementary Table S2). Previous reports indicated that some naturally occurring mutations in Nef could affect both SERINC3 and SERINC5 (N51T, H116N, V148L/X, 161N and M168I/X), while others only affected SERINC3 (8R, 9S, 11P, 12G, 14A, 15A, 21K, 28E, 43I, 182E and 188S) or SERINC5 (14S, 21R, 54D, 63E, 81F, 120F, 157N and 158K) [14,25,26]. The change of charge in the protein interaction site (AA 12–39) and the presence of amino acids 12G, N51T, H116N and 188S in Nef were associated with a significant increased infectivity of Bal26-pseudotyped HIV-1 produced in the presence of SERINC3, while increased length of the protein interaction site (AA12–39), 9S, 43I and S163C also increased and 11P, 14A, V148X, 161N and R178G decreased infectivity, albeit not significantly (Figure 5A and Supplementary Table S2). A change of charge in the protein interaction site (AA 12–39) and the S163C mutation in the ExxxLL binding motif of Nef increased, whereas the loss of valine at position 148 (V148X) decreased, the HIV-1 infectivity of Bal26-pseudotyped HIV-1 produced in the presence of SERINC5 (Figure 5B and Supplementary Table S2). Nef variants containing 21R and H116N also increased infectivity, whereas the variant R178G decreased infectivity, albeit not significantly (Figure 5B and Supplementary Table S2).

### 3.4. Effect of Naturally Occurring Mutations in Nef on Disease Progression

**Figure 6:**
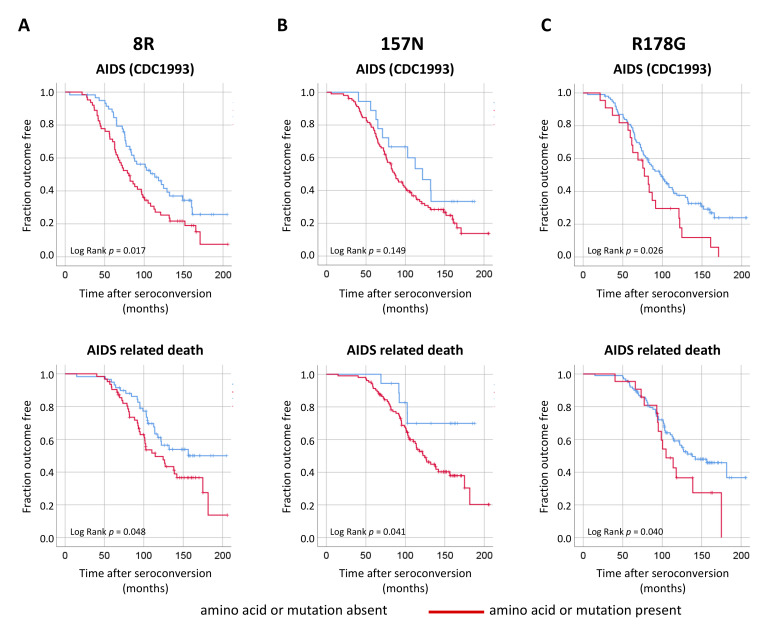
Effect of naturally occurring mutations in Nef on disease progression. Kaplan–Meier survival analysis for amino acid variations or mutations 8R (**A**), 157N (**B**) and R178G (**C**) in Nef with time in months from seroconversion to progression to AIDS as defined by the CDC definition 1993 (top panel) or AIDS-related death (bottom panel). *P* values displayed represent log-rank test.

The effect of the naturally occurring mutations in Nef on HIV-1 disease progression was determined by Kaplan–Meier and Cox proportional hazard survival analyses using AIDS according to the 1993 CDC definition or AIDS-related death (Figure 6 and Supplementary Table S3) as endpoints. We observed that individuals with HIV-1 containing the 8R, 157N or R178G polymorphism in Nef showed an accelerated disease progression. Individuals with an HIV-1 variant containing arginine at position 8 (8R) in Nef progressed after AIDS (log rank, *p* = 0.017; relative hazard (RH), 1.66; 95% CI, 1.09–2.55; *p* = 0.018), asparagine at position 157 (157N) in Nef died earlier from AIDS-related disease (log rank, *p* = 0.041; RH, 2.52; 95% CI, 1.00–6.31; *p* = 0.048) and the R178G Nef mutation showed faster progression to AIDS (log rank, *p* = 0.026; RH, 1.78; 95% CI, 1.06–2.97; *p* = 0.037) and AIDS-related death (log rank, *p* = 0.040; RH, 1.84; 95% CI, 1.00–3.37; *p* = 0.048) (Figure 6 and Supplementary Table S3).

## 4. Discussion

Nef is a multifunctional viral protein that has the ability to downregulate cell surface molecules, including CD4, MHC-I and T-cell receptor [1], allowing HIV-1 to evade ADCC and CTLs [2,3,4,5]. Nef can also alter the activation state of T cells and macrophages and perturbs the actin cytoskeleton by engaging with cellular kinases [6,7,8]. In the present study, we evaluated the effect of naturally occurring mutations in HIV-1 Nef on its ability to counteract SERINC3 and SERINC5 restriction and disease progression. Our analysis showed that, in agreement with previous studies HIV-1, Nef was able to counteract the restriction of SERINC3 and SERINC5 in vitro and thereby enhanced HIV-1 infectivity. We observed high variability in the primary Nef amino acid sequences and also demonstrated variability in their ability to downregulate SERINC3 as well as SERINC5. Most Nef proteins showed an increased activity to counteract SERINC3 and SERINC5 as compared to NL4-3 Nef, indicating an important role for Nef in vivo.

Different regions and amino acid polymorphisms in the Nef protein have been described to affect the ability to counteract SERINC3- and SERINC5-mediated downregulation. Residues 12 to 39, which are located in the N-terminal, largely unfolded anchor domain of Nef, were recently implicated in a wide range of Nef activities. The residues were originally described to recruit the Nef-associated kinase complex (NAKC) to facilitate HIV-1 replication in primary target cells [7,27] and enhance the secretion of extracellular vesicles containing Nef and proinflammatory cytokines [28,29]. Interestingly, it was shown that deletions in this protein interaction site of HIV-1_SF2_ Nef prevent the downregulation of CD4 and MHC-I and the antagonism of SERINC5 [30,31]. Moreover, this protein interaction site contains the amino acid stretch RxRxRR on position 17–22, which was shown to be involved in the anchoring of Nef to the membrane and exosome release [32,33,34,35]. In primary HIV-1 variants, we observed variation in the length and the number of positively charged amino acids in this region (protein interaction site). Our in vitro analysis showed that the change in the number of positively charged amino acids in this region was associated with increased ability of Nef to counteract SERINC3 and SERINC5. However, no effect of the charge changes was observed on HIV-1 disease progression. Several specific amino acids located in and near the protein interaction site in Nef have been described to affect the ability of Nef to downregulate SERINC3/5 [26]. We observed that glycine at position 12 and serine at position 188 of Nef were associated with increased infectivity, indicating an increased ability to downregulate SERINC3 and confirming previous observations [26]. However, the effect of the other mutations in this region or in close proximity (AA8-43) on either SERINC3 and/or SERINC5 downregulation could not be confirmed. This can most likely be explained by the high variability (including length and charge) of the protein interaction site in Nef obtained from primary HIV-1 variants, possibly containing additional mutations compensating for the loss or gain of Nef function. Arginine at position 8 in this region was shown to have an effect on disease progression. The 8R has previously been associated with loss of the ability to internalize SERINC3 in subtype A, C and D HIV-1 and, in combination with glycine at position 11, poor activity to internalize SERINC3 in subtype B HIV-1; however, 8R had no effect on SERINC5, CD4 and MHC class I downregulation [26]. In our cohort, this amino acid was present in 52% of the individuals with HIV-1 and was shown to be associated with an accelerated disease progression, which cannot be explained by a loss of the ability to counteract SERINC3 and indicates that this amino acid has another effect in vivo and, for instance, allows immune escape from cytotoxic T cells or compensates for other attenuating (escape) mutations.

Nef confiscates the endocytic and late secretory pathways through the modulation of adapter and accessory proteins involved in vesicle formation, such as the AP complexes and dynamin [4,36,37,38]. Nef interrupts MHC-I trafficking by binding to MHC-I in the endoplasmic reticulum or early Golgi, after which the AP-1 protein is recruited in the *trans*-Golgi network (Nef:MHC-I:AP-1 tripartite complex) [39,40,41,42,43,44,45,46,47,48]. AP-1 directs MHC-I, subsequently, into the endo-lysosomal pathway [43,44,49]. In contrast, CD4 trafficking is not inhibited by Nef. Instead, Nef internalizes CD4 at the plasma membrane [49,50,51,52,53]. The downregulation of CD4 by Nef was shown to require AP-2 (Nef:AP-2:CD4 ternary complex). This ternary complex at the plasma membrane has been proposed to promote clathrin-dependent endocytosis [54,55,56]. In addition, the AP-1 protein was shown to be involved in Nef-dependent CD28 downregulation [57]; however, an earlier study also demonstrated involvement of the AP-2 protein [58]. Downregulation of SERINC3/5 by Nef is also thought to require clathrin and AP-2 to redistribute these proteins to the endosomes [9,10,59].

Nef has conserved sequences, such as dileucine motifs (ExxxLL; Figure 1), the diacidic motif (DD174-175) and the three charged C-terminal residues (ERE177; Figure 1) in the C-loop to interact or to help establish an interaction with the endocytic machinery, mainly AP-1 and AP-2 proteins [15,16,50,60,61,62]. These motifs are necessary for the downregulation of CD4 and, in the case of the ExxxLL motif, also MHC-I [4,15]. In addition, Nef requires these dileucine and diacidic motifs to counteract SERINC5 activity by sending it to the endosomes [31]. It was shown that although Nef is one of the most variable proteins amongst primate lentiviruses, the dileucine motif is highly conserved between species because it is required to abolish SERINC5 activity [31,63]. Previously, an amino acid polymorphism (S163) in the ExxxLL region was associated with the ability of Nef to downregulate CD4 [64]. We observed that this polymorphism (S163C) was present in 39.8% of the primary HIV-1 variants tested and was associated with increased Nef activity to counteract SERINC5 and, consequently, with higher HIV-1 infectivity. This is in agreement with an earlier study by Jin et al. demonstrating higher activity against SERINC5 of Nef containing cysteine at position 163 [14]. The 157N is located near the ExxxLL region and has previously been demonstrated to have a slightly decreased ability to internalize SERINC5 as compared to Nef that does not have asparagine at this position [26]. Although we did not observe an effect of this mutation in the context of primary Nef variants on infectivity of HIV-1 produced in the presence of SERINC3 and SERINC5, the asparagine at position 157 in Nef was associated with faster disease progression. This may indicate that this mutation is associated with escape from the immune system and, thus, contributes to disease progression.

The R178G polymorphism located in the ERE177 region was observed in almost 18% of the primary isolates. Our in vitro analysis showed that this mutation lowered HIV-1 infectivity, albeit not significantly, indicating a possible loss of the ability of these Nef proteins to counteract SERINC3 and SERINC5. Interestingly, the presence of the R178G mutation in Nef was associated with accelerated progression to AIDS and AIDS-related death in our cohort. The lower ability of the R178G mutation to counteract SERINC3 and -5 may indicate that, during the course of infection, the selective pressure of this restriction protein is lost in these individuals, explaining the selective outgrowth of HIV-1 variants containing the R178G mutation in Nef. A recent study delivered strong evidence for the role of R178 in AP-2 recruitment, showing direct interaction of this amino acid with a pocket formed by the AP-2 α:σ2 subunit hemicomplex [16,65], and therefore, this mutation may indeed affect the ability of Nef to counteract SERINC3 and -5. Indeed, the mutation of the three charged C-terminal residues in the C-loop of Nef (ERE177AAA), including R178, abrogated the function of Nef to counteract SERINC5 [63], which confirms our in vitro observation. However, the charged residues ERE177 were also found to be crucial for CD4 downmodulation, but not for MHC-I downmodulation, and enhanced HIV-1 infectivity [15,16,42]. A recent study also showed that the G176R mutation in Nef selectively disrupts CD4 downregulation, but not SERINC5 antagonism by Nef, likely by disruption of the AP-2 binding and the uncoupling of these two functions of Nef [62]. These data could also indicate that the observed accelerated disease progression associated with the R178G mutation may be related to the loss of the ability of Nef to downregulate CD4. Downregulation of CD4 is thought to be important for HIV-1 replication and pathogenesis because it prevents the premature death of HIV-1-infected cells by coinfection or the induction of apoptosis via CD4 signaling [66,67,68,69,70]. Therefore, the accelerated disease progression may also be explained by an increased loss of infected CD4 cells, especially in the gut-associated lymphoid tissues where a high number of infected CD4 cells reside [71], which, consequently, may result in increased bacterial translocation and high immune activation [72,73].

Several other amino acid polymorphisms in Nef have been identified to play a role in SERINC3 or SERINC5 antagonism [14,25,26]. In our study, we did not find a significant effect of most of these mutations on Nef activity against SERINC3 or SERINC5. However, we did observe that the N51T, H116N and 188S mutations increased HIV-1 infectivity when the virus was produced in the presence of SERINC3. The H116N mutation has been reported to have a negative effect on infectivity in the presence of SERINC5 but had no effect on SERINC3 internalization [14,26], contradicting our observations. However, no effect of the H116N mutation in Nef on disease progression was observed, indicating that the change in functionality associated with this polymorphism is not essential or is too small to affect HIV-1 replication in vivo. Serine at position 188 has previously been described with increased SERINC3 internalization function [26], confirming our observations, while the N51T mutation has only been associated with a modest increased functionality to internalize SERINC5 [14]. In agreement with our observation, the loss of valine at position 148 has been associated with the loss of the ability to counteract SERINC5 [14] and lower infectivity. However, no effect of these mutations on disease progression was observed.

Here, we observed that amino acid changes in primary Nef affect the ability of this protein to counteract SERINC3- and SERINC5-mediated HIV-1 restriction. However, these mutations, except for 8R, 157N and R178G, have no significant effect on HIV-1 pathogenesis. Nef activity was determined in vitro in the presence of SERINC3 and SERINC5 overexpression, and this may not be reflective of Nef function in vivo where SERINC3 and SERINC5 expression may be lower and Nef proteins with lower in vitro antagonizing activity might still be able to sufficiently maintain viral infectivity. It is, therefore, likely that the Nef function to counteract SERINC3 and SERINC5 is maintained during viral evolution in vivo, similar to its ability to downregulate CD4 and MHC class I.

## Figures and Tables

**Table 1:** Amino acid variation in HIV-1 obtained from individuals with HIV-1.

Nef Variation		% of Patients (*n*) *n* = 123	
Protein interaction site (AA 12–39):			
Extra length		46.3% (57)	
Change of charge		55.3% (68)	
Amino acid polymorphisms:			
8R	52.0% (64)	81F	10.6% (13)
9S	63.4% (78)	H116N	17.9% (22)
11P	5.7% (7)	120F	6.5% (8)
12G	82.1% (101)	V148L/V148X	10.6% (13)/13.8% (17)
14A/14P	5.7% (7)/25.2% (31)	157N	85.4% (105)
15A	52.8% (65)	158K	11.4% (14)
21K/21R	11.4% (14)/64.2% (79)	161N	93.5% (115)
28E	35.8% (44)	S163C	39.8% (49)
43I	82.1% (101)	M168I/M168X	22.0% (27)/27.6% (34)
N51T	61.8% (76)	R178G	17.9% (22)
54D	78.0% (96)	182E	19.5% (24)
63E	79.7% (98)	188S	6.5% (8)

## Data Availability

The data presented in this study are available in the article and supplementary material.

## Supplementary Materials

The following are available online at https://www.mdpi.com/1999-4915/13/3/423/s1, Table S1: Amino acid variations in Nef proteins obtained from individuals with HIV-1; Table S2: Mean HIV-1 infectivity in the presence of SERINC3/5 in the absence or presence of naturally occurring mutations in Nef; Table S3: Cox Regression analysis for progression to AIDS and AIDS-related death.
