# Supplementary material for: Nef Obtained from Individuals with HIV-1 Vary in Their Ability to Antagonize SERINC3- and SERINC5-Mediated HIV-1 Restriction
Source: Viruses. 2021 Mar 6;13(3):423. doi: 10.3390/v13030423 (PMC8000780; doi:10.3390/v13030423)
Supplement: Supplementary file 1 [file viruses-13-00423-s001.pdf]

Supplementary Table S1. Amino acid variations in Nefs obtained from HIV-1 infected individuals.

Patient ID	Positively charged AA (AA 12-39)	Extra length (AA 12-39)	Amino Acid position																								
			8	9	11	12	14	15	21	24	28	43	51	54	63	81	116	120	148	157	158	161	163	168	178	182	188
19999	0	0	R	C	A	G	S	T	K	E	E	I	T	D	E	Y	N	Y	V	N	E	N	V	M	K	Q	R
19984	0	0	I	-	V	G	S	N	R	E	V	I	S	A	E	Y	N	Y	V	N	E	N	N	M	R	E	R
19981	0	4	R	S	G	G	S	A	R	E	V	I	T	D	D	F	H	Y	A	N	E	N	C	M	R	V	R
19980	0	4	C	-	S	G	P	T	R	E	A	I	T	T	E	Y	N	Y	V	N	E	N	S	I	G	V	R
19974	0	0	C	-	V	G	P	K	E	E	D	V	T	D	D	Y	H	Y	V	N	E	N	C	M	G	K	K
19970	4	9	R	N	E	G	D	T	K	E	E	I	T	D	D	F	I	Y	V	N	A	N	A/C	M	G	M	S
19962	-1	0	R	S	S	Q	P	A	Q	E	D	I	N	A	E	Y	N	Y	V	N	E	N	S	M	K	Q	R
19961	0	0	C	K	G	G	S	T	R	E	D	I	T	D	E	Y	H	Y	V	N	E	N	T	M	R	E	R
19956	-1	0	C	S	V	G	P	T	R	E	D	I	N	D	D	Y	H	Y	V	N	K	N	S	M	R	E	R
19954	0	0	R	S	V	G	P	T	R	E	E	I	N	A	E	Y	H	Y	V	N	K	N	S	M	R	K	R
19951	0	4	R	S	G	G	P	A	Q	K	E	V	T	D	E	Y	H	Y	L	N	E	N	S	L	R	V	R
19947	-2	0	R	S	-	G	E	A	E	E	D	V	N	D	E	F	H	Y	V	N	E	N	C	I	K	V	S
19937	-1	4	R	S	E	G	S	A	Q	E	E	V	T	D	E	Y	H	Y	L	N	E	N	C	M	K	V	R
19933	-2	0	R	S	N	E	P	A	E	E	E	I	T	D	E	Y	N	Y	V	N	E	N	S	I	K	V	R
19932	-1	0	R	-	R	E	S	V	Q	E	-	I	T	D	E	Y	H	Y	V	N	E	N	S	M	G	M	R
19922	-1	4	R	-	R	E	T	A	H	E	E	I	T	D	E	Y	H	Y	V	N	E	N	S	M	R	M	R
19918	4	12	S	N	G	G	A	A	R	P	E	I	T	D	E	W	H	Y	V	N	E	N	C	I	G	M	R
19902	0	4	C	S	-	G	S	A	R	E	A	I	N	A	D	F	H	Y	I	T	E	N	S	M	R	M	S
19900	1	4	Y	-	S	G	S	T	R	R	E	I	T	D	E	Y	H	Y	V	N	E	N	S	M	K	V	H
19899	0	4	C	S	-	G	P	A	E	E	E	I	T	A	E	F	H	Y	V	N	E	N	C	M	G	Q	R
19887	0	4	C	S	V	G	P	A	K	E	V	I	T	D	E	Y	H	F	V	N	E	N	R	M	R	V	T
19869	4	10	R	K	V	D	P	T	R	E	D	I	T	D	E	Y	H	F	V	N	E	N	S	M	G	M	M
19866	-1	4	R	M	-	E	T	A	Q	E	V	I	T	D	E	Y	H	Y	L	N	E	N	S	I	R	V	R
19861	0	4	S	S	V	G	S	A	R	E	E	I	N	D	D	Y	H	Y	A	N	E	N	C	M	R	M	R
19858	-1	4	R	-	R	E	T	V	Q	E	E	I	T	D	E	Y	H	Y	V	N	E	N	C	I	R	M	R
19843	-1	0	R	R	P	G	P	A	K	E	E	L	N	D	E	Y	H	Y	V	N	K	N	C	I	K	E	K

19841	0	0	R	M	-	E	S	T	R	E	D	I	T	D	E	Y	N	Y	V	N	E	N	C	M	G	V	H
19832	0	0	S	S	V	G	P	T	R	E	D	I	T	A	E	Y	H	Y	V	N	K	N	C	V	R	E	R
19831	-1	0	S	S	V	G	P	A	Q	E	D	L	N	A	E	Y	H	Y	V	N	E	N	C	M	K	M	R
19830	-1	0	S	S	V	G	P	A	R	E	D	I	T	D	E	Y	H	Y	V	N	E	N	C	M	R	V	S
19828	-1	0	S	S	V	G	P	A	R	E	D	I	T	D	E	Y	H	Y	V	N	E	N	C	M	R	V	S
19814	0	0	R	S	I	G	P	T	R	E	D	I	T	D	E	Y	H	Y	V	N	E	N	S	M	R	Q	R
19801	2	4	L	-	R	G	S	A	E	E	D	I	T	D	E	Y	N	Y	V	N	E	N	S	M	K	Q	R
19793	2	10	C	S	G	G	P	K	R	D	D	L	N	A	E	Y	H	Y	V	N	A	N	S	M	G	M	L
19792	-1	0	R	-	R	E	S	V	Q	E	V	I	T	D	E	Y	H	Y	V	N	E	N	S	M	G	M	R
19789	1	0	C	S	G	G	H	T	R	E	D	I	N	A	E	Y	N	Y	V	N	K	K	S	I	G	E	R
19784	0	0	S	S	I	G	P	A	K	E	V	V	T	D	E	F	N	Y	V	N	E	T	C	M	R	M	S
19776	0	0	S	S	V	G	P	T	R	E	E	I	S	D	E	Y	H	Y	V	N	K	N	C	M	G	Q	R
19764	0	0	S	S	F	G	P	T	R	E	D	I	T	D	E	Y	H	Y	V	N	E	N	R	M	G	Q	R
19761	-1	0	R	S	G	G	P	A	Q	E	E	I	N	A	E	Y	H	Y	V	N	E	N	C	M	G	M	R
19742	-1	0	R	S	G	G	P	A	I	E	D	I	T	D	E	S	H	Y	V	N	E	N	C	M	K	Q	R
19730	0	0	R	S	F	G	P	A	K	E	E	I	T	A	E	Y	H	Y	V	N	E	N	C	M	R	V	R
19728	-2	0	C	S	G	G	A	A	R	E	V	L	N	A	D	Y	H	Y	L	N	E	N	C	I	R	V	R
19722	0	4	M	-	V	G	S	T	R	E	A	I	T	D	E	W	H	Y	V	N	E	N	C	M	K	M	R
19700	4	16	R	I	G	G	P	A	R	E	E	I	T	D	E	Y	H	Y	L	N	K	N	S	M	R	V	R
19689	-1	4	C	-	V	G	P	T	R	E	D	I	T	D	E	Y	N	Y	V	N	E	N	S	M	R	E	R
19684	0	5	R	S	A	G	P	A	R	E	E	I	N	D	E	Y	H	Y	V	N	E	N	T	M	K	Q	R
19680	0	0	S	S	I	G	P	T	K	E	D	I	T	D	D	Y	H	Y	V	N	E	N	S	M	R	E	R
19663	-1	0	R	S	N	E	P	A	E	E	E	V	T	D	D	Y	H	Y	V	T	E	N	C	M	K	V	R
19659	0	4	C	S	-	G	P	A	R	E	V	I	T	D	E	Y	H	Y	V	N	K	N	S	M	R	E	R
19655	0	0	S	S	V	G	R	A	R	E	E	I	N	D	E	Y	H	Y	V	N	E	N	C	M	K	M	H
19646	0	0	S	S	I	G	P	A	K	E	E	I	T	D	E	Y	H	Y	V	N	E	N	S	I	R	V	R
19644	-1	0	R	N	D	G	F	N	R	E	D	I	N	D	E	L	K	Y	V	D	E	K	S	M	R	E	S
19642	0	0	S	S	V	G	P	A	R	E	D	L	N	D	E	Y	H	F	V	N	K	N	R	M	K	Q	S
19629	1	8	R	L	N	Q	S	T	R	G	V	I	T	D	D	Y	H	Y	V	T	E	N	C	M	R	Q	R
19628	0	0	S	S	V	G	P	A	K	E	E	I	N	A	E	Y	H	Y	V	N	E	N	S	M	R	V	R
19617	-1	0	R	-	R	E	A	A	Q	E	E	I	T	D	E	Y	H	Y	V	N	E	N	S	M	K	V	R
19607	-1	4	R	K	N	E	P	A	E	P	D	I	N	D	D	W	H	Y	V	N	E	N	S	M	K	V	R

19593	0	2	S	R	F	G	S	T	R	E	D	V	T	D	E	Y	H	Y	V	N	A	N	S	I	K	Q	R
19589	5	12	R	S	G	G	R	A	R	V	D	I	T	D	E	Y	N	Y	V	N	E	N	C	M	K	A	H
19576	0	4	R	M	G	G	S	T	R	G	D	I	N	D	E	Y	H	Y	V	N	E	N	C	I	K	M	R
19574	0	3	S	S	I	G	P	T	R	P	D	I	T	D	E	Y	H	Y	V	N	K	N	S	M	R	Q	R
19566	5	9	Y	-	S	G	S	T	R	R	E	I	T	D	E	Y	H	Y	V	N	E	N	S	M	K	V	H
19559	0	0	S	S	I	G	P	T	R	E	D	I	S	D	D	Y	H	Y	V	T	E	N	R	I	K	E	R
19554	-1	4	L	S	R	G	T	A	R	E	D	I	N	D	D	Y	H	Y	V	N	E	N	C	I	R	V	R
19550	-1	0	R	S	G	G	A	S	I	E	D	I	N	T	D	Y	H	Y	I	T	E	N	C	I	K	V	R
19545	-2	0	R	S	G	G	A	A	R	D	D	I	N	D	E	Y	H	Y	V	N	E	N	C	I	R	M	R
19544	0	0	R	S	A	G	P	A	R	E	E	I	N	A	E	H	H	Y	V	N	K	N	C	M	R	E	R
19537	2	0	R	S	D	G	H	R	R	E	V	I	T	D	D	Y	H	Y	V	N	E	N	S	M	R	V	
19528	-1	0	C	S	V	G	P	T	R	E	D	I	N	D	E	Y	H	Y	V	N	E	N	C	M	R	M	R
19514	0	0	R	K	F	E	P	A	R	E	E	I	T	D	D	Y	H	Y	V	N	E	N	S	M	K	V	R
19510	3	10	R	S	G	G	P	A	R	E	E	I	T	D	E	Y	N	Y	V	N	N	N	S	R	R	E	R
19500	1	0	H	S	V	G	P	K	R	E	E	I	T	A	E	Y	H	Y	V	N	K	N	C	M	K	E	R
19489	1	11	R	S	I	G	P	A	R	E	D	I	N	D	E	H	H	Y	L	S	E	N	T	M	R	V	L
19463	2	14	R	S	G	G	P	A	R	Q	A	I	N	D	E	Y	H	F	V	N	E	N	S	M	R	V	R
19455	2	8	S	S	I	G	P	A	R	K	D	I	T	D	E	Y	H	Y	V	N	E	N	Y	M	R	E	R
19453	1	4	R	S	S	G	S	T	R	E	E	I	N	A	E	Y	H	Y	V	N	E	N	S	M	R	V	R
19443	0	2	R	V	-	G	S	T	R	E	E	I	T	D	E	Y	H	Y	V	N	E	N	C	M	K	V	R
19429	-1	2	R	S	G	G	S	T	R	E	D	I	T	A	D	H	H	Y	V	N	E	N	S	I	K	M	S
19428	0	0	C	S	-	-	S	A	E	E	D	V	N	D	E	Y	H	Y	V	N	A	N	S	M	R	Q	R
19424	0	0	-	-	A	G	P	K	A	E	V	I	T	D	D	F	H	Y	V	N	E	N	C	M	K	Q	R
19419	-3	0	-	-	S	A	P	A	Q	D	E	T	N	A	E	Y	N	Y	L	A	D	N	S	M	K	M	R
19417	0	4	R	S	G	G	P	T	R	E	D	I	T	D	D	Y	N	Y	V	N	E	N	S	V	R	V	H
19415	0	0	L	G	-	-	P	T	R	E	D	I	S	D	E	Y	H	Y	V	N	E	N	S	M	R	E	R
19412	4	7	S	S	V	G	P	R	R	D	E	I	T	D	E	Y	N	Y	V	T	E	D	C	M	R	Q	S
19400	-1	0	R	S	I	E	P	A	T	E	D	L	N	D	D	F	H	Y	V	N	E	N	S	M	K	V	R
19385	-1	0	R	S	A	G	P	A	E	E	D	I	T	D	E	H	H	Y	L	N	E	N	S	M	K	V	T
19383	0	0	C	-	A	G	P	A	R	E	D	I	T	D	E	Y	H	Y	V	N	K	N	S	M	R	E	R
19342	0	0	R	-	P	G	P	A	R	E	D	I	T	D	E	Y	H	F	V	N	E	N	T	I	R	M	R
19335	0	0	I	V	-	-	S	A	R	E	D	I	N	D	D	Y	H	Y	V	N	E	N	S	M	R	E	R

19334	-1	0	R	S	G	G	S	A	E	E	E	L	N	A	D	Y	H	Y	V	N	E	N	S	M	G	M	R
19332	0	0	-	S	V	G	P	A	K	E	E	I	T	D	E	Y	H	Y	V	N	E	N	C	M	K	V	K
19329	-1	0	H	K	G	G	P	A	Q	E	D	I	N	A	E	Y	H	F	V	N	E	N	C	M	K	V	R
19322	0	0	R	S	G	G	A	A	R	E	D	I	T	D	E	Y	H	Y	M	T	S	N	C	I	K	M	H
19310	1	0	S	S	V	G	P	K	R	D	D	I	N	D	D	Y	H	Y	V	N	E	N	T	M	R	E	R
19308	-1	4	R	M	-	Q	T	V	Q	E	E	I	T	D	E	Y	H	Y	V	N	E	N	S	I	G	M	R
19298	3	8	P	S	A	G	S	T	R	E	D	I	T	D	E	Y	H	Y	V	N	E	N	S	M	G	M	R
19296	0	0	R	S	F	G	P	T	R	E	A	I	N	D	E	Y	H	Y	V	N	E	N	S	M	R	E	P
19285	1	4	K	S	V	G	P	K	R	E	D	I	N	A	E	Y	N	Y	V	N	K	N	S	M	K	K	T
19266	0	3	R	S	V	G	P	A	K	E	V	L	T	D	E	Y	H	Y	V	N	E	T	S	M	K	V	K
19250	-2	4	H	S	R	G	E	A	R	E	E	L	N	A	E	F	H	Y	L	T	E	N	C	L	R	I	R
18976	0	0	S	S	G	G	P	A	K	E	-	I	N	D	E	Y	H	Y	V	N	E	D	C	M	R	M	R
18971	4	13	S	S	V	G	A	N	R	E	A	L	T	D	D	Y	H	Y	V	N	E	N	C	M	K	T	R
18969	1	6	S	S	V	G	P	A	K	E	D	I	T	D	E	Y	H	Y	L	N	Q	N	S	M	K	I	S
18965	-2	0	R	R	G	G	S	A	Q	E	E	I	T	D	D	F	H	Y	L	N	E	N	S	M	K	I	R
18942	0	4	R	K	V	D	P	T	R	E	D	I	T	D	E	F	H	Y	V	N	E	N	S	M	G	Q	R
18932	-1	5	N	K	A	G	P	A	R	E	E	I	T	D	E	Y	N	Y	V	T	E	N	S	M	G	Q	R
18919	-1	2	R	S	V	G	P	A	R	E	V	I	T	D	E	F	H	Y	V	N	E	N	S	M	G	M	R
18909	0	5	R	G	S	G	P	A	R	E	A	I	N	A	E	Y	H	Y	V	N	E	N	C	M	R	M	R
18905	0	0	R	S	G	G	S	A	R	E	-	I	N	D	E	Y	N	F	V	N	G	N	C	M	K	Q	R
18888	-1	1	R	V	G	G	P	A	E	E	E	V	T	D	E	Y	H	F	V	N	E	N	S	I	G	Q	R
18887	1	4	R	S	P	G	S	E	R	R	E	I	T	D	E	Y	N	Y	V	N	E	N	S	M	K	E	R
18880	0	4	R	S	P	G	S	V	R	A	V	I	T	D	E	Y	N	Y	V	T	E	N	C	I	K	V	R
18865	2	7	N	S	A	G	S	R	R	D	E	I	T	D	E	Y	N	Y	V	T	E	T	C	I	R	Q	S
18860	-2	0	R	I	G	G	E	T	R	-	D	L	N	D	E	Y	H	Y	L	T	E	N	C	I	R	V	R
18839	2	3	R	S	P	G	S	T	R	R	V	I	N	D	E	Y	H	Y	V	T	T	T	C	I	N	E	R
18829	1	0	H	S	-	G	P	R	R	E	E	I	T	A	E	Y	H	Y	V	N	E	N	C	M	K	Q	R
18818	0	3	R	S	P	G	S	T	R	E	V	I	T	D	E	F	H	Y	L	N	E	N	S	L	K	V	K
18814	0	0	R	S	P	G	S	T	K	E	D	I	T	D	E	Y	H	Y	V	N	E	N	S	I	R	V	R
18806	6	13	N	-	N	G	S	V	R	P	D	L	N	D	E	Y	H	Y	V	K	E	N	C	I	R	C	A
18789	0	0	R	I	G	G	P	A	R	E	E	I	T	D	E	Y	H	Y	V	N	E	N	N	M	K	M	R
11679	0	0	R	S	G	G	P	T	R	E	D	I	T	D	E	Y	N	Y	V	N	E	N	S	M	K	E	S

11668	0	0	S	S	G	R	H	A	R	E	E	I	T	A	E	Y	H	Y	V	N	E	N	S	M	G	E	R

	Infectivity in the presence of			
	SERINC3 (mean ±σ)		SERINC5 (mean ±σ)	
Nef variation	absent	present	absent	present
Extra length (AA 12-39)	1.98 ± 1.2	3.16 ± 2.6	2.98 ± 3.2	3.16 ± 2.1
Change of charge (AA 12-39)	**1.78 ± 1.4***	**3.03 ± 2.3***	**2.10 ± 1.7***	**3.65 ± 3.0***
N51T	**1.85 ± 1.1***	**3.56 ± 2.7***	2.72 ± 3.0	3.55 ± 2.1
H116N	**2.04 ± 1.3****	**6.23 ± 2.7****	2.82 ± 2.6	4.81 ± 2.7
V148X	3.11 ± 2.2	1.63 ± 0.7	**3.82 ± 2.8***	**1.65 ± 0.9***
S163C	2.22 ± 1.9	2.80 ± 2.2	**2.23 ± 1.7***	**3.69 ± 3.1***
M168X	3.17 ± 2.3	2.46 ± 1.8	4.01 ± 3.2	2.72 ± 1.5
R178G	2.94 ± 2.2	1.80 ± 1.4	3.25 ± 3.0	2.71 ± 2.0
8R	2.77 ± 2.0	2.95 ± 2.2	4.31 ± 3.8	3.06 ± 1.9
9S	2.29 ± 0.8	3.14 ± 2.4	3.24 ± 1.6	3.61 ± 3.1
11P	2.99 ± 2.2	1.99 ± 0.6	3.58 ± 2.8	2.76 ± 1.6
12G	**1.85 ± 2.0***	**3.07 ± 2.1***	2.56 ± 1.3	3.66 ± 2.9
14S	2.81± 2.1	3.59 ± 2.4	3.54 ± 2.8	3.10 ± 1.3
14A	2.99 ± 2.2	1.92 ± 0.5	3.68 ± 2.8	1.85 ± 1.0
15A	2.68 ± 1.9	3.28 ± 2.5	3.48 ± 2.9	3.53 ± 2.4
21K	2.85 ± 2.2	3.12 ± 1.6	3.61 ± 2.9	2.84 ± 1.7
21R	2.34 ± 1.4	3.56 ± 2.6	2.88 ± 1.5	4.25 ± 3.6
28E	3.01 ± 2.3	2.66 ± 1.8	3.88 ± 3.1	2.78 ± 1.5
43I	1.90 ± 0.7	3.22 ± 2.3	2.57 ± 2.0	3.81 ± 2.9
54D	2.66 ± 2.0	4.96 ± 2.4	3.50 ± 2.8	3.48 ± 1.8
63E	2.93 ± 1.6	2.86 ± 2.4	2.95 ± 1.4	3.79 ± 3.2

81F	2.89 ± 2.3	2.84 ± 0.9	3.58 ± 2.9	3.04 ± 1.1
120F	3.04 ± 2.2	2.04 ± 0.9	3.53 ± 2.9	3.31 ± 1.4
157N	2.87 ± 2.0	2.89 ± 2.2	3.62 ± 1.4	3.46 ± 3.1
158K	2.73 ± 2.1	3.77 ± 2.0	3.54 ± 2.9	3.27 ± 1.9
161N	3.91 ± 2.7	2.77 ± 2.0	4.08 ± 0.9	3.43 ± 2.9
182E	3.01 ± 2.3	2.21 ± 0.6	3.54 ± 2.9	3.25 ± 1.9
188S	**2.68 ± 2.1***	**4.76 ± 1.8***	3.42 ± 2.9	4.15 ± 0.9

Supplementary Table S2. Mean HIV-1 infectivity in the presence of SERINC3/5 in the absence or presence of natural occurring mutations in Nef.

*$p < 0.05$. **$p < 0.005$

Supplementary Table S3. Cox Regression analysis for progression to AIDS and AIDS related death.

	AIDS (CDC1993)		AIDS related death	
Nef AA variant	*p*-value	Exp(B) (95.0% CI)	*p*-value	Exp(B) (95.0% CI)
N51T	0.438	1.18 (0.76-1.83)	0.499	1.20 (0.70-2.03)
H116N	0.190	0.68 (0.38-1.20)	0.125	0.55 (0.26-1.17)
V148X	0.304	1.33 (0.76-2.33)	0.922	1.03 (0.52-2.03)
S163C	0.966	0.99 (0.64-1.51)	0.923	0.97 (0.58-1.62)
M168X	0.711	1.09 (0.68-1.72)	0.568	1.16 (0.68-1.99)
R178G	**0.037**	**1.78 (1.06-2.97)**	**0.048**	**1.84 (1.00-3.37)**
8R	**0.018**	**1.66 (1.09-2.55)**	0.051	1.66 (0.99-2.76)
9S	0.610	0.89 (0.58-1.37)	0.348	1.29 (0.75-2.20)
11P	0.602	0.76 (0.28-2.09)	0.426	0.56 (0.13-2.31)
12G	0.370	1.30 (0.73-2.31)	0.883	1.04 (0.55-1.97)
14A	0.774	1.14 (0.46-2.82)	0.644	1.27 (0.45-3.51)
14S	0.182	1.37 (0.86-2.20)	0.352	1.31 (0.74-2.32)
15A	0.512	0.86 (0.57-1.32)	0.793	0.93 (0.56-1.54)
21K	0.674	0.86 (0.44-1.67)	0.196	0.54 (0.21-1.36)
21R	0.918	1.02 (0.66-1.58)	0.622	0.88 (0.52-1.46)
28E	0.937	0.98 (0.63-1.51)	0.425	0.80 (0.47-1.36)
43I	0.070	1.72 (0.95-3.11)	0.323	1.40 (0.71-2.77)
54D	0.815	1.06 (0.63-1.78)	0.429	1.30 (0.67-2.49)
63E	0.681	0.89 (0.53-1.50)	0.275	0.72 (0.40-1.29)
81F	0.632	1.16 (0.62-2.19)	0.974	0.98 (0.44-2.16)
120F	0.872	1.07 (0.46-2.45)	0.675	1.21 (0.48-3.03)
157N	0.152	1.58 (0.84-2.99)	**0.048**	**2.52 (1.00-6.31)**

158K	0.865	0.94 (0.50-1.78)	0.219	0.56 (0.22-1.40)
161N	0.122	2.21 (0.80-6.05)	0.081	5.81 (0.80-41.99)
182E	0.773	0.92 (0.55-1.55)	0.265	0.68 (0.34-1.34)
188S	0.958	0.97 (0.42-2.24)	0.697	0.81 (0.29-2.25)
